# Invasive group A streptococcal infections requiring admission to ICU: a nationwide, multicenter, retrospective study (ISTRE study)

**DOI:** 10.1186/s13054-023-04774-2

**Published:** 2024-01-02

**Authors:** Arthur Orieux, Renaud Prevel, Margot Dumery, Jean-Baptiste Lascarrou, Noémie Zucman, Florian Reizine, Pierre Fillatre, Charles Detollenaere, Cédric Darreau, Nadiejda Antier, Mélanie Saint-Léger, Guillaume Schnell, Béatrice La Combe, Charlotte Guesdon, Franklin Bruna, Antoine Guillon, Caroline Varillon, Olivier Lesieur, Hubert Grand, Benjamin Bertrand, Shidasp Siami, Pierre Oudeville, Céline Besnard, Romain Persichini, Pierrick Bauduin, Martial Thyrault, Mathieu Evrard, David Schnell, Johann Auchabie, Adrien Auvet, Jean-Philippe Rigaud, Pascal Beuret, Maxime Leclerc, Asaël Berger, Omar Ben Hadj Salem, Julien Lorber, Annabelle Stoclin, Olivier Guisset, Léa Bientz, Pierre Khan, Vivien Guillotin, Jean-Claude Lacherade, Alexandre Boyer, ISTRE Group, Arthur Orieux, Renaud Prevel, Margot Dumery, Jean-Baptiste Lascarrou, Noémie Zucman, Florian Reizine, Pierre Fillatre, Charles Detollenaere, Cédric Darreau, Nadiejda Antier, Mélanie Saint-Léger, Guillaume Schnell, Béatrice La Combe, Charlotte Guesdon, Franklin Bruna, Antoine Guillon, Caroline Varillon, Olivier Lesieur, Hubert Grand, Benjamin Bertrand, Shidasp Siami, Pierre Oudeville, Céline Besnard, Romain Persichini, Pierrick Bauduin, Martial Thyrault, Mathieu Evrard, David Schnell, Johann Auchabie, Adrien Auvet, Jean-Philippe Rigaud, Pascal Beuret, Maxime Leclerc, Asaël Berger, Omar Ben Hadj Salem, Julien Lorber, Annabelle Stoclin, Olivier Guisset, Léa Bientz, Pierre Khan, Vivien Guillotin, Jean-Claude Lacherade, Alexandre Boyer

**Affiliations:** 1 Service de Médecine Intensive Réanimation, Hôpital Pellegrin et Hôpital Saint André, CHU de Bordeaux, Place Amélie Raba Léon, 33000 Bordeaux, France; 2 Unité INSERM U1045, Université de Bordeaux, Bordeaux, France; 3 Service de Médecine Intensive Réanimation, CHU de Nantes, Nantes, France; 4 Service de Réanimation Médico-Chirurgicale, CH Annecy Genevois, Epagny Metz-Tessy, France; 5 Service de Réanimation Polyvalente, CH de Vannes, Vannes, France; 6 Service de Réanimation Polyvalente, CH de Saint Brieuc, Saint Brieuc, France; 7 Service de Réanimation – Unité de Soins Continus, CH de Boulogne Sur Mer, Boulogne, France; 8 Service de Réanimation Médico-Chirurgicale, CH Le Mans, Le Mans, France; 9 Service de Réanimation, CH Alès – Cévennes, Alès, France; 10 Service de Réanimation, CH Périgueux, Périgueux, France; 11 Service de Réanimation Médico-Chirurgicale, Groupe Hospitalier du Havre, Le Havre, France; 12 Service de Réanimation Polyvalente, Groupe Hospitalier Bretagne Sud, Lorient, France; 13 Service de Réanimation Polyvalente, Centre Hospitalier de Pau, Pau, France; 14 Service de Réanimation, CH Alpes Leman, Contamine Sur Arve, France; 15 Service de Médecine Intensive Réanimation, INSERM, Centre d’Étude des Pathologies Respiratoires (CEPR), UMR 1100, CHRU de Tours, Université de Tours, Tours, France; 16 Service de Médecine Intensive Réanimation, CH Dunkirk, Dunkirk, France; 17 Service de Réanimation Médico-Chirurgical, CH La Rochelle, La Rochelle, France; 18 Service de Réanimation Polyvalente, Hôpital Robert Boulin, Libourne, France; 19 Service de Réanimation Polyvalente, CH Intercommunal Toulon, La Seyne sur Mer (CHITS), Toulon, France; 20 Service de Réanimation Polyvalente, CH Sud Essonne, Étampes, France; 21 Service de Réanimation Médicale, Groupe Hospitalier Régional Mulhouse Sud Alsace (GHRMSA), Mulhouse, France; 22 Service de Médecine Intensive Réanimation, CH Régional de Orléans, Orléans, France; 23 Service de Réanimation Et Soins Continus, CH de Saintonge, Saintes, France; 24 Service de Médecine Intensive Réanimation, CHU de Caen, Caen, France; 25 Service de Réanimation Polyvalente, Groupe Hospitalier Nord Essonne – site Longjumeau, Longjumeau, France; 26 Service Réanimation Polyvalente et Surveillance Continue, CH de Lens, Lens, France; 27 Service de Réanimation Polyvalente, CH d’Angoulême, Angoulême, France; 28 Service de Réanimation Polyvalente, CH de Cholet, Cholet, France; 29 Service de Réanimation Polyvalente, CH de Dax, Dax, France; 30 Service de Médecine Intensive Réanimation, CH de Dieppe, Dieppe, France; 31 Service de Réanimation et Soins Continus, CH de Roanne, Roanne, France; 32 Service de Réanimation et Soins Intensifs Polyvalents, CH Mémorial Saint-Lô, Saint-Lô, France; 33 Service de Réanimation, CH de Haguenau, Haguenau, France; 34 Service de Réanimation Médico-Chirurgicale, CHI Meulan – les Mureaux, Meulan en Yvelines, France; 35 Service de Médecine Intensive Réanimation, CH de Saint Nazaire, Saint Nazaire, France; 36 Département Interdisciplinaire d’Organisation des Parcours Patients (DIOPP), Service de Réanimation, Gustave Roussy Cancer Campus, Villejuif, France; 37 Laboratoire de Bactériologie, CHU de Bordeaux; Microbiologie Fondamentale et Pathogénicité UMR5234, Université de Bordeaux, Bordeaux, France; 38 Département d’Anesthésie Réanimation Sud, Centre Médico-Chirurgical Magellan, Hôpital Haut Lévêque, CHU de Bordeaux, Pessac, France; 39 Service de Médecine Intensive Réanimation, CH Départemental de la Vendée, La Roche-sur-Yon, France

**Keywords:** Invasive group A streptococcal infection, Streptococcal toxic shock syndrome intensive care unit, ICU mortality

## Abstract

**Background:**

Group A *Streptococcus* is responsible for severe and potentially lethal invasive conditions requiring intensive care unit (ICU) admission, such as streptococcal toxic shock-like syndrome (STSS). A rebound of invasive group A streptococcal (iGAS) infection after COVID-19-associated barrier measures has been observed in children. Several intensivists of French adult ICUs have reported similar bedside impressions without objective data. We aimed to compare the incidence of iGAS infection before and after the COVID-19 pandemic, describe iGAS patients’ characteristics, and determine ICU mortality associated factors.

**Methods:**

We performed a retrospective multicenter cohort study in 37 French ICUs, including all patients admitted for iGAS infections for two periods: two years before period (October 2018 to March 2019 and October 2019 to March 2020) and a one-year after period (October 2022 to March 2023) COVID-19 pandemic. iGAS infection was defined by Group A *Streptococcus* isolation from a normally sterile site. iGAS infections were identified using the International Classification of Diseases and confirmed with each center's microbiology laboratory databases. The incidence of iGAS infections was expressed in case rate.

**Results:**

Two hundred and twenty-two patients were admitted to ICU for iGAS infections: 73 before and 149 after COVID-19 pandemic. Their case rate during the period before and after COVID-19 pandemic was 205 and 949/100,000 ICU admissions, respectively (*p* < 0.001), with more frequent STSS after the COVID-19 pandemic (61% vs. 45%, *p* = 0.015). iGAS patients (*n* = 222) had a median SOFA score of 8 (5–13), invasive mechanical ventilation and norepinephrine in 61% and 74% of patients. ICU mortality in iGAS patients was 19% (14% before and 22% after COVID-19 pandemic; *p* = 0.135). In multivariate analysis, invasive mechanical ventilation (OR = 6.08 (1.71–21.60), *p* = 0.005), STSS (OR = 5.75 (1.71–19.22), *p* = 0.005), acute kidney injury (OR = 4.85 (1.05–22.42), *p* = 0.043), immunosuppression (OR = 4.02 (1.03–15.59), *p* = 0.044), and diabetes (OR = 3.92 (1.42–10.79), *p* = 0.008) were significantly associated with ICU mortality.

**Conclusion:**

The incidence of iGAS infections requiring ICU admission increased by 4 to 5 after the COVID-19 pandemic. After the COVID-19 pandemic, the rate of STSS was higher, with no significant increase in ICU mortality rate.

**Supplementary Information:**

The online version contains supplementary material available at 10.1186/s13054-023-04774-2.

## Background

*Streptococcus pyogenes,* also called group A *Streptococcus* (GAS), is a Gram-positive bacterium responsible for human diseases ranging from pauci-symptomatic superficial infections (pharyngitis, impetigo) to severe and potentially lethal, invasive conditions (necrotizing fasciitis or streptococcal toxic shock-like syndrome [STSS]) named invasive group A streptococcal (iGAS) infections [1]. In 2005, the World Health Organization (WHO) reported an estimated 18 million cases of severe GAS infections and more than 500,000 deaths per year [2]. More severe iGAS infections require admission to intensive care unit (ICU) and are associated with a high mortality rate (more than 50% in STSS patients) [2].

The risk factors for iGAS infection are numerous. Diabetes, cardiac disease, and acute kidney injury (AKI) are associated with an increased risk of invasive GAS disease [3–5]. Also, children, the immunocompromised (HIV infection, malignancy), and older patients are at the most significant risk of iGAS infections. Moreover, the management of iGAS infections is debated. Early surgical source control (debridement of necrotic tissue, surgical drainage) is a vital management part [6]. The use of adjunctive clindamycin may improve outcomes because of its antitoxin effects and excellent tissue penetration, even in patients without STSS or necrotizing fasciitis [7]. At present, there is a lack of data about intravenous immunoglobulin (IVIG) use and consensus about linezolid for the treatment of iGAS infection [8, 9].

Since September 2022, European countries and the United States have been affected by increased pediatric cases of iGAS infections and pediatric iGAS infections requiring admission to ICU [10, 11]. Still, most reported cases, whether they are associated with STSS or not, are superinfections of viral (influenza or respiratory syncytial virus [RSV]) respiratory infections, as previously reported [12, 13]. It seems to result from a rebound after barrier measures in children whose immune system has not been in contact with commonly circulating GAS strains [14].

After the COVID-19 pandemic, a Danish study [15] reported an abnormally high incidence of GAS meningitis in adults (11 cases) along with another study describing 4 cases of severe iGAS infections, some of which in adults, in Nederland [16].

Recently, several intensivists of French adult ICUs reported concerns about an increase in iGAS infection incidence, reporting mostly skin and soft tissue infections (SSTI) and lower respiratory tract infections (LRTI). On March 2023, the French Public Health Agency published in its report a relative stability of severe iGAS infections in adults but an increase in the frequency of STSS and LRTIs linked to GAS in this population [14].

Nevertheless, to date, no objective data support this bedside impression. The ISTRE (Infections invasives à STreptocoque du groupe A en RÉanimation) study aims to compare the incidence of iGAS infection requiring admission to the ICU before and after the COVID-19 pandemic. It also aims to describe iGAS patients’ characteristics, treatments, and outcomes and determine associated factors with ICU mortality.

## Methods

### Study design

We conducted a retrospective multicenter study in 37 ICUs in France. Of 89 ICUs in metropolitan France that were invited to participate in the ISTRE retrospective multicenter study, 37 accepted (Additional file 2: Fig. S1). The patients’ data were routinely collected in dedicated electronic health records during their hospital stay. Enrollment and collection of hospitalization dates were completed retrospectively. The manuscript was prepared according to the STROBE guidelines for observational studies.

### Patients

Patients were enrolled if they were 18 years or older, admitted to ICU and developed confirmed iGAS infection for three 6-month periods (October 1st to March 31st): 2 periods before (2018–2019 and 2019–2020) and 1 period after (2022–2023) the COVID-19 pandemic. No statistical sample size was calculated in the absence of relevant data to predict the outcome. However, anticipating that fewer iGAS infection would have occurred in the pre-COVID-19 period compared to the post-COVID-19 period, and to increase the statistical power of the analysis, we included patients over two 6-month periods in the before COVID-19 study period. iGAS infections were identified by searching the hospital databases for codes A.40.0 and B.95.0 in the International Classification of Diseases-10th revision and were confirmed with the microbiology laboratory databases in each center. iGAS infections were defined by GAS isolation from a normally sterile site (blood, CSF, joint fluid, pleural fluid, peritoneal fluid, tissue, urine, intra-operative pus or internal swab). There were no exclusion criteria. For patients with multiple ICU admissions, only the first ICU stay was considered.

The incidence of iGAS infections was expressed in case rate or person-time rate [17] (number of incident events divided by the cumulative at-risk time in the sample: a time-constant incidence hazard): number of iGAS infections during period of interest divided by 100,000 ICU admissions for period [18].

### Clinical variables and outcomes

Immunosuppression was defined as chronic use of immunosuppressive drugs, corticosteroid therapy (≥ 2 mg/kg per day or ≥ 20 mg daily of prednisone for more than 14 days), cancer or hematologic malignancy, and chronic kidney disease (CKD) as an estimated glomerular filtration rate (GFR) of < 60 mL/min/1.73m^2^, persisting for at least 3 months corresponding to CKD stage 3 or more according to the KDIGO classification [19].

Patients’ characteristics on 24 first hours of ICU admission included age, gender, body mass index, chronic comorbidities (e.g., hypertension, diabetes mellitus, stroke, immunosuppression, ischemic cardiomyopathy, chronic obstructive pulmonary disease (COPD) and asthma, CKD, alcohol and tobacco use, and non-steroidal anti-inflammatory drugs (NSAID).

Patients’ presentation was characterized by the Sequential Organ Failure Assessment (SOFA) score calculated based on the worst clinical and laboratory values recorded on Day 1, STSS according to the Working Group on Severe Streptococcal Infections definition [20] (Additional file 1), septic shock according to Third International Consensus Definitions [21], AKI occurrence (using the worst serum creatinine or diuresis) during the ICU stay and minimum diuresis defined by the minimum diuresis/kg/h according to KDIGO classification, and routine laboratory parameters [22]. The day of ICU admission is considered day 0, and the following calendar day is day 1.

Patients’ care was recorded from ICU admission to discharge: antimicrobial therapy, surgical source control requirement, life-sustaining therapies including invasive mechanical ventilation (IMV), vasoactive drugs (norepinephrine and dobutamine), renal replacement therapy (RRT), and veno-arterial extracorporeal membrane oxygenation (VA-ECMO).

Patients’ outcomes consisted of ICU and hospital length of stay and mortalities.

### Statistical analysis

Descriptive statistics included mean ± standard deviation (SD) or median [interquartile range (IQR)] if the variable did not fit a normal distribution. Quantitative variables were compared using a Mann–Whitney test. Qualitative variables were compared using Fisher’s exact test.

Univariate comparison was performed using linear logistic regression (all variables fit normal distribution). Independent variables with a *p* value < 0.20 in the univariate analysis were considered for inclusion in the multivariate analysis using linear logistic regression.

For both regression analyses, we excluded the “renal replacement therapy” variable due to collinearity with the “acute kidney injury” variable.

We applied a backward stepwise selection for period, age, diabetes, immunosuppression, infection site, STSS, invasive mechanical ventilation, dobutamine use and acute kidney injury to identify factors significantly associated with ICU mortality in iGAS patients.

We applied a backward stepwise selection for age, diabetes, immunosuppression, infection site, clindamycin use, invasive mechanical ventilation, and acute kidney injury to identify factors significantly associated with ICU mortality in STSS patients.

The ratio was of one independent variable for eight events. Goodness-of-fit of regression analyses and multicollinearity detection were presented in Additional file 1.

To estimate ICU mortality’s hazard ratio (HR) between STSS patients and non-STSS patients and between pre- and post-COVID-19 periods, we used standard Cox regression model. We assessed and confirmed the proportional hazard assumption with log(− log) plots and the scaled Schoenfeld residuals (Additional file 1).

A value of *p* < 0.05 was considered statistically significant (double-sided).

Statistical analysis was carried out using Jamovi (The Jamovi project 2022, Version 2.3) and R (version 4.2.3, R Foundation for Statistical Computing, Vienna, Austria).

### Ethics

According to French law, the database was notified to our Data Protection Officer. The study obtained the approval of the ethics committee of the French Intensive Care Society (#CE SRLF 23-004). Patients (or their relatives, if any) were notified about the anonymized use of their health care data by the department’s booklet and an individual information letter addressed to the patient or his relatives, in accordance with French law on retrospective studies of anonymized data. This study complied with French research Reference Methodology MR004 regarding health-data privacy and the French National Commission on Informatics and Liberty (CNIL).

## Results

### Incidence of iGAS infection

**Fig. 1:**
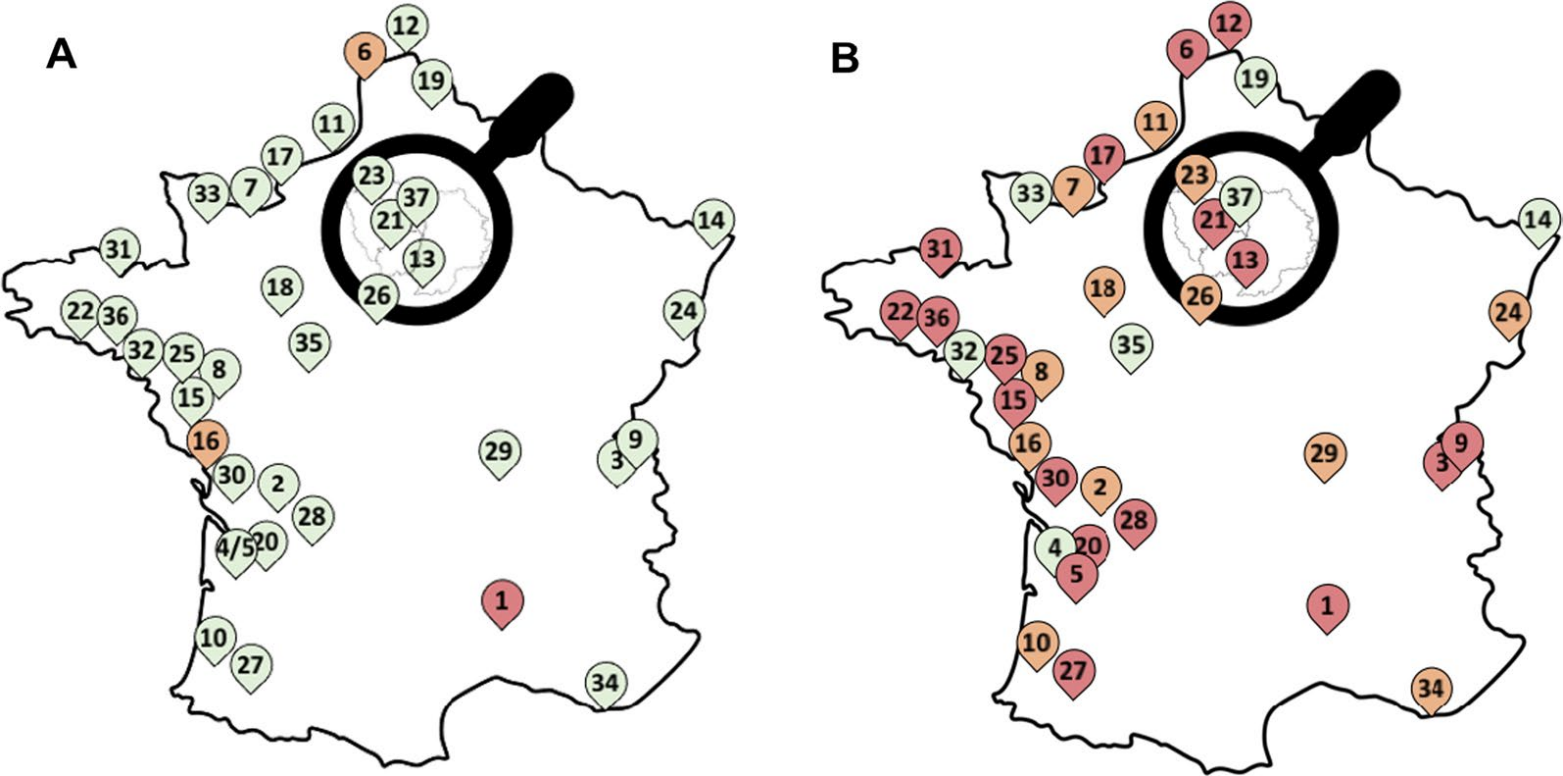
iGAS infections case rate in metropolitan’s ICUs before and after COVID-19. Case rate > 1000: red, Case rate 500–1000: orange, Case rate < 500: green. **A** Before COVID-19 (October 2018 to March 2019 and October 2019 to March 2020). **B** After COVID-19 (October 2022 to March 2023)

Two hundred and twenty-two patients were admitted to ICU for iGAS infections. Seventy-three patients out of 35,610 ICU admissions (0.20%) and 149 out of 15,963 ICU admissions (0.95%) were hospitalized for severe iGAS disease before and after the COVID-19 pandemic, respectively (Additional file 2: Table S1). Overall, the case rate of iGAS infection was 205 and 949/100,000 ICU admissions, respectively (*p* < 0.001) (Fig. 1 and Additional file 2: Table S1). Details of ICU admissions, iGAS infections, and case rates according to the study period for each center are presented in Additional file 2: Table S1.

### Baseline characteristics of iGAS patients

The 37 participating ICUs were distributed throughout metropolitan France: six (19%) in university hospitals and 31 (81%) in non-university hospitals (Additional file 2: Fig. S1). Detailed patients’ characteristics are presented in Table 1. Briefly, the median age was 61 [43–70], most patients were men (58%), 80 (36%) of them had a history of hypertension and 40 (19%) of diabetes. The median SOFA score at ICU admission was 8 [5–13]. iGAS infections caused SSTI in 44% of cases and LRTI (pneumonia, empyema or abscess) in 34% of cases.

One hundred and twenty-three patients (55%) had STSS. STSS was more common after than before COVID-19 (61% vs. 45%, respectively, *p* = 0.015).

Influenza co-infection was less frequent before the COVID-19 pandemic (*p* < 0.001), whereas less hypertension (*p* = 0.010), coronary disease (*p* = 0.011), COPD (*p* < 0.001), CKD (*p* = 0.003) and alcohol use disorders (*p* = 0.014) were observed in the post-pandemic period.

**Table 1:** Baseline characteristics of patients at ICU admission

Baseline characteristics	All patients (*n* = 222)	Before COVID-19 (*n* = 73)	After COVID-19 (n = 149)	*p* value
Male, *n* (%)	128 (58%)	41 (59%)	87 (58%)	0.753
Age (years), *median (IQR)*	61 (43–70)	62 (49–70)	60 (42–69)	0.265
BMI (kg/m^2^), *median (IQR)*	26 (22.9–30.9)	26 (23.3–32.3)	25.9 (22.9–30.5)	0.643
Hypertension, *n* (%)	80 (36%)	35 (48%)	45 (30%)	0.010
Diabetes, *n* (%)	40 (18%)	16 (22%)	24 (13%)	0.290
Coronary disease, *n* (%)	23 (10%)	13 (18%)	10 (7%)	0.011
Immunosuppression, *n* (%)	16 (7%)	7 (10%)	9 (6%)	0.337
Asthma, *n* (%)	18 (8%)	5 (7%)	13 (8%)	0.631
COPD, *n* (%)	13 (6%)	11 (15%)	2 (1%)	< 0.001
Chronic kidney disease, *n* (%)	14 (6%)	9 (12%)	5 (3%)	0.003
Stroke, *n* (%)	10 (5%)	3 (4%)	7 (5%)	0.843
Active tobacco use, *n* (%)	51 (23%)	22 (30%)	29 (19%)	0.076
Alcohol use disorders, *n* (%)	33 (15%)	17 (23%)	16 (11%)	0.014
Prior NSAID use,* n* (%)	31 (14%)	11 (15%)	20 (13%)	0.740
Infection sites				0.144
*Skin and soft tissue, n* (%)	98 (44%)	37 (51%)	60 (40%)	
*Lower respiratory tract. n* (%)	75 (34%)	18 (25%)	57 (38%)	
*Others, n* (%)	49 (22%)	16 (22%)	33 (22%)	
SOFA score at D1, *median (IQR)*	8 (5–13)	7 (5–10)	9 (6–13)	0.011
*Cardiovascular, median (IQR)*	4 (1–4)	4 (1–4)	4 (1–4)	
*Renal, median (IQR)*	2 (0–3)	1 (0–3)	2 (0–3)	
*Respiration, median (IQR)*	2 (1–3)	2 (0.5–2)	2 (1–3)	
*Coagulation, median (IQR)*	1 (0–2)	0 (0–1)	1 (0–2)	
*Central nervous system, median (IQR)*	0 (0–1)	0 (0–1)	0 (0–1)	
*Liver, median (IQR)*	0 (0–1)	0 (0–1)	0 (0–1)	
STSS,* n* (%)	123 (55%)	32 (45%)	91 (61%)	0.015
*STSS with renal manifestations, n (%)*	119 (54%)	33 (45%)	86 (58%)	
*STSS with liver manifestations, n (%)*	88 (40%)	20 (27%)	68 (46%)	
*STSS with ARDS, n (%)*	79 (36%)	15 (21%)	64 (43%)	
*STSS with soft tissue necrosis, n (%)*	75 (34%)	22 (30%)	53 (36%)	
*STSS with coagulopathy manifestations, n (%)*	75 (34%)	11 (15%)	64 (43%)	
Acute kidney injury,* n* (%)	155 (70%)	47 (64%)	108 (76%)	0.217
Ferritin (ng/mL)º, *median (IQR)*	603 (371–1490)	403 (312–597)	763 (419–1704)	0.013
Fibrinogen (mg/L)^$^, *median (IQR)*	6.6 ± 2	6.5 ± 2.1	6.7 ± 2.0	0.445
Lymphocyte count (G/L)^¤^, *median (IQR)*	0.37 (0.19–0.64)	0.35 (0.21–0.61)	0.37 (0.18–0.70)	0.804
Neutrophil count (G/L)^§^, *mean* ± *SD*	11.1 ± 8.3	13.8 ± 7.8	9.7 ± 8.2	< 0.001
Albumin (g/L)^‡^, *mean* ± *SD*	23.4 ± 6.8	22.9 ± 6.6	24 ± 7.1	0.284
COVID-19 co-infection,* n* (%)	5 (2%)	0 (0%)	5 (3%)	0.113
Influenza co-infection,* n* (%)	33 (15%)	2 (3%)	31 (21%)	< 0.001
Others active viral co-infection,* n* (%)	8 (4%)	1 (1%)	7 (5%)	0.211

Statistical significance (*p* < 0.05)

*ARDS* acute respiratory distress syndrome, *BMI* body mass index, *COPD* chronic obstructive pulmonary disease, *D* day, *ENT* ear, nose, throat, *ICU* intensive care unit, *IQR* interquartile range, *NSAID* non-steroidal anti-inflammatory drug, *SD* standard deviation, *SOFA score* Sequential Organ Failure Assessment Score, *STSS* streptococcal toxic shock syndrome

Others infection sites include orthopedic, ear nose and throat, gynecological, neurologic, abdominal, heart, urologic and vascular infections

SOFA scores can range from 0 (no organ failure) to 24 (most severe level of multiorgan failure)

º *n* = 187; ^$^ *n* = 184; ^¤^ *n* = 210; ^§^ *n* = 215; ^¥^ *n* = 215; ^‡^ *n* = 175

### Treatment of iGAS patients

Detailed patients’ care is provided in Table 2. The first antibiotic regimen prescribed in the emergency department or in the ICU was active against GAS in 211 (99%) patients. Early empiric antimicrobial treatment was initiated without delay at ICU admission in all the 222 (100%) patients. The median time from symptom onset to effective antibiotic therapy was 0 [0–0] day. An active monotherapy was used in 64 patients (29%), 131 patients (59%) received double-therapy and 26 patients (12%) had an active triple-therapy against GAS. Amoxicillin was used in 110 patients (50%), 78 patients (35%) received third generation cephalosporins, and piperacillin/tazobactam was prescribed in 42 (19%) patients and aminoglycosides in 40 patients (18%). Clindamycin and linezolid were used in 124 (56%) and 34 patients (15%), respectively, and clindamycin use increased after COVID-19 pandemic (44% before and 63% after COVID-19; *p* = 0.001). Intravenous immunoglobulin (IVIG) was prescribed in 17 patients (8%) (in addition to clindamycin in 15 out of 17 patients). Surgical treatment was required in 90 patients (41%) with a median time from ICU admission to surgery of 0 [0–1] day.

Mechanical ventilation and norepinephrine were required in 135 (61%) and 164 (74%) of patients, respectively, with increase after COVID-19 pandemic both for the proportion of patients receiving IMV (*p* = 0.014) and the maximum norepinephrine dose required (*p* = 0.002). Twenty-three (11%) and 3 patients (1%) required dobutamine and VA-ECMO support, respectively. One hundred fifty-five out of 222 patients (70%) suffered from AKI during ICU hospitalization and 57 (26%) required RRT.

**Table 2:** Treatment and outcomes of iGAS infections

	All patients (*n* = 222)	Before COVID-19 (*n* = 73)	After COVID-19 (*n* = 149)	*p* value
***Treatment of iGAS infections***				
Time between onset of symptoms and ICU admission (days), *median (IQR)*	3 (1–4)	2 (1–5)	3 (1–4)	0.438
Time between ICU admission and iGAS infection confirmed (days), *median (IQR)*	0 (0–1)	0 (0–1)	0 (0–1)	0.235
Empiric antimicrobial therapy at ICU admission,* n* (%)	221 (100%)	72 (99%)	149 (100%)	1
Time from ICU admission to effective antimicrobial therapy (days), *median (IQR)*	0 (0–0)	0 (0–0)	0 (0–0)	0.235
*Monotherapy, n (%)*	64 (29%)	30 (41%)	34 (23%)	0.005
*Double therapy, n (%)*	131 (59%)	38 (52%)	93 (62%)	0.140
*Triple therapy, n (%)*	26 (12%)	5 (7%)	21 (14%)	0.115
Clindamycin use,* n* (%)	124 (56%)	30 (44%)	94 (63%)	0.001
Linezolid use,* n* (%)	34 (15%)	6 (6%)	28 (19%)	0.040
Intravenous immunoglobulin use,* n* (%)	17 (8%)	2 (3%)	15 (10%)	0.054
Surgical source control needed,* n* (%)	90 (41%)	27 (37%)	63 (42%)	0.450
Time from ICU admission to surgical source control (days), *median (IQR)*	0 (0–1)	0 (0–1)	0 (0–1)	0.965
Invasive mechanical ventilation, *n* (%)	135 (61%)	36 (49%)	99 (66%)	0.014
Length of intubation (days), *median (IQR)*	7 (2–14)	6 (3–15)	8 (2–14)	0.998
Norepinephrine use, *n* (%)	164 (74%)	50 (68%)	114 (77%)	0.173
Maximum norepinephrine dose (μg/kg/min), *median (IQR)*	0.9 (0.4–2)	0.6 (0.2–1.2)	1.2 (0.5–2.1)	0.002
Dobutamine use, *n* (%)	23 (11%)	4 (5%)	19 (14%)	0.065
Renal replacement therapy, *n* (%)	57 (26%)	17 (23%)	40 (29%)	0.569
Veno-arterial ECMO, *n* (%)	3 (1%)	1 (1%)	2 (1%)	0.987
***Outcome of iGAS infections***				
ICU length of stay (days), *median (IQR)*	7 (4–16)	6 (4–16)	8 (4–16)	0.278
Hospitalization length of stay (days), *median (IQR)*	22 (11–35)	23 (9–37)	22 (13–34)	0.711
ICU death, n (%)	43 (19%)	10 (14%)	33 (22%)	0.135
Deaths at day 90, *n* (%)	46 (21%)	13 (18%)	33 (22%)	0.820
*Time from ICU admission to death (days), median (IQR)*	2 (0–7)	1 (1–3)	2 (1–13)	0.540

Statistical significance (*p* < 0.05)

*ECMO* extracorporeal membrane oxygenation, *ICU* intensive care unit, *iGAS* invasive group A streptococcal, *IQR* interquartile range

### Outcomes of iGAS patients

Patients’ outcomes are detailed in Table 2. Median ICU length of stay was 7 [4–16] days and median hospital length of stay 22 [11–35] days. Forty-three out of 222 patients (19%) deceased in ICU and 46 (22%) at day 90, with a median time from ICU admission to death of 2 [0–7] days. iGAS mortality in ICU was 14% before and 22% after COVID-19 pandemic (*p* = 0.135). iGAS mortality at day 90 was 18% before and 22% after COVID-19 pandemic (*p* = 0.82) (Additional file 2: Fig. S2).

### Factors associated with ICU mortality in iGAS patients

In univariate analysis, age (OR = 1.03 [1.01–1.06], *p* = 0.005), diabetes (OR = 2.23 [1.01–4.91], *p* = 0.047), STSS (OR = 11.03 [3.78–32.14]), *p* < 0.001), IMV (OR = 8.43 [2.89–24.58], *p* < 0.001), dobutamine use (OR = 2.88 [1.16–7.11], *p* = 0.022), AKI (OR = 11.69 [2.74–49.91], *p* < 0.001) and RRT (OR = 4.90 [2.42–9.94], *p* < 0.001) were significantly associated with ICU mortality (Additional file 2: Table S2). Of note, study period (after *vs* before COVID-19 pandemic) was not associated with ICU mortality (univariate analysis, OR = 1.79 [0.93–3.87], *p* = 0.138).

In multivariate analysis, IMV (OR = 6.08 [1.71–21.60], *p* = 0.005), STSS (OR = 5.75 [1.71–19.22], *p* = 0.005), AKI (OR = 4.85 [1.05–22.42], *p* = 0.043), immunosuppression (OR = 4.02 [1.03–15.59], *p* = 0.044), and diabetes (OR = 3.92 [1.42–10.79], *p* = 0.008) remained significantly associated with ICU mortality (Table 3).

**Table 3:** Factors associated with ICU mortality for iGAS infections

Variables	Multivariate analysis		
	OR	CI95%	*p* value
Diabetes	3.92	1.42–10.79	0.008
Immunosuppression	4.02	1.03–15.59	0.044
STSS	5.75	1.71–19.22	0.005
Invasive mechanical ventilation	6.08	1.71–21.60	0.005
Acute kidney injury	4.85	1.05–22.42	0.043

Statistical significance (*p* < 0.05)

*CI confidence interval, ICU* intensive care unit, *OR odds ratio, STSS* streptococcal toxic shock syndrome

Univariate comparison was performed using linear logistic regression (Table S1). Independent variables with a *p* value < 0.20 were considered for inclusion in the multivariate analysis using linear logistic regression. We excluded “renal replacement therapy” variable due to collinearity with “acute kidney injury” variable. We applied a backward stepwise selection for period, age, diabetes, immunosuppression, infection site, STSS, invasive mechanical ventilation, dobutamine use and acute kidney injury to identify factors significantly associated with ICU mortality in iGAS patients. The ratio was of one independent variable for 8 events

### Mortality in STSS patients (*n* = 123)

**Fig. 2:**
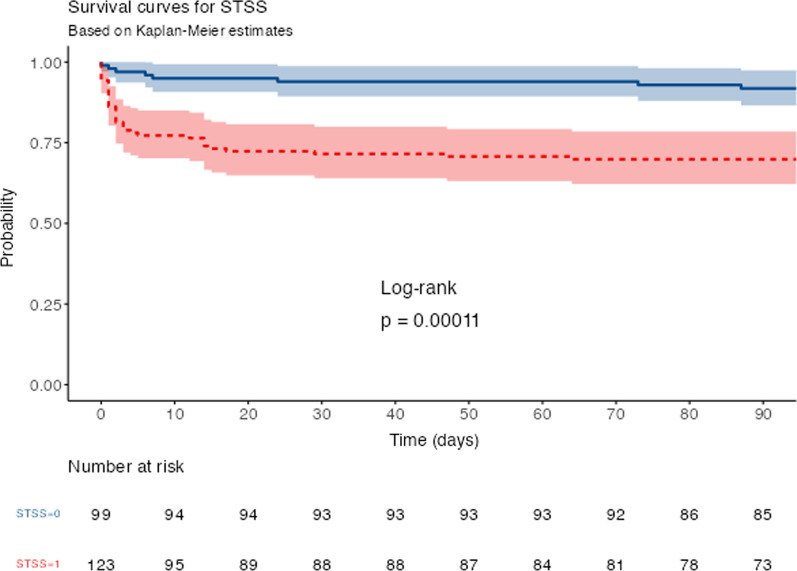
Comparison of survival rates according to time from iGAS infection with and without streptococcal toxic shock syndrome (STSS)

The ICU and day 90 mortality rates were superior in the STSS group than in the non-STSS group (32% vs. 4%, *p* < 0.001 and 33% versus 6%, *p* < 0.001, respectively), and STSS was associated with an increased hazard for ICU mortality (HR 9.19 [3.29–25.76]; *p* < 0.001) (Fig. 2).

### Factors associated with ICU mortality in STSS patients

Univariate and multivariate analysis were reported in Additional file 2: Table S3. In multivariate analysis, IMV (OR = 19.60 [2.52–152.53], *p* = 0.005), AKI (OR = 11.58 [1.34–99.78], *p* = 0.026), and diabetes (OR = 8.79 [2.26–34.21], *p* = 0.002) were significantly associated with increased ICU mortality, while clindamycin use was associated with a decreased ICU mortality (OR = 0.20 [0.08–0.54], *p* = 0.002).

## Discussion

Incidence of iGAS requiring admission to ICU increased after COVID-19 pandemic in our study. Main clinical presentations were skin and soft tissue infections (SSTI) and lower respiratory tract infections (LRTI).

Interestingly, critical iGAS patients suffered from fewer comorbidities and exhibited a higher frequency of influenza co-infection after than before the pandemic. After the COVID-19 pandemic, patients suffered from more severe iGAS infection (more frequent STSS, higher SOFA score at ICU admission, IMV requirement, and higher maximal dose of norepinephrine). Nevertheless, ICU mortality was not different after than before the COVID-19 pandemic.

The increase in iGAS incidence is consistent with recent data from UK pediatric wards published in 2023, which found that iGAS is a significant and increasing cause of severe bacterial infection, with a significant burden of mortality and persistent morbidity [23]. SSTI and LRTI were the predominant sites of infection, similar to our data. Regarding adults, risk factors for iGAS were consistent with previously described data, i.e., tobacco, alcohol abuse, wounds or chronic skin lesions, homelessness, and diabetes [24].

A first explanation for this increased incidence of critical iGAS in adults could be the emergence of a highly virulent GAS strain. Isolated GAS strains are routinely sent to the French National Reference Center in case of invasive infections, and GAS is then classified based on the sequence of the 5′ end of the gene encoding the M protein (*emm*) with more than 220 *emm* described genotypes [25]. The recent European epidemic of GAS infection seems to be linked to the increase in the frequency of an already known genotypes (emm-1 with sequence-type 28, and emm-12) [14, 26–28]. Interestingly, a Belgian study reported an increase in the proportion of the toxigenic M1UK lineage [29] with an increased expression of the superantigen gene spA [30] compared to the original M1global lineage.

Nevertheless, this “bacterial hypothesis” could only be one part of the explanation. This increase in iGAS incidence occurs in patients with fewer comorbidities (hypertension, coronary disease, COPD, CKD and alcohol abuse disorder) but with a higher proportion of active influenza co-infection, which raises “the host hypothesis” as a complementary explanation. Indeed, seasonal variation has previously been observed with increased iGAS incidence during winter, coinciding with the influenza season [31, 32]. The severity of viral respiratory infections can worsen in case of simultaneous (co-infection) or subsequent (superinfection) bacterial infection. The interaction of influenza A virus with another *Streptococcus* specie has been more extensively investigated, demonstrating that pneumococcal infections are more severe (increased pneumococcal burden, lung inflammation, and mortality) in mice after non-lethal influenza A virus infection [33]. Regarding GAS, some data suggest a similar increase in bacterial infection occurring after influenza [31].

A decrease in exposure to pathogens after two years of barrier measures (social isolation, hand hygiene, cloth face masks) due to the COVID-19 pandemic could be responsible for a loss of immune system sensitivity and also explain the increase in iGAS infections after COVID-19 [34].

It could have been hypothesized that our results were due to the health system overwhelming and health workers’ exhaustion after the COVID-19 pandemic [35, 36]. However, our study observed no delay in ICU admission, treatment initiation, and surgical source control if needed. After the COVID-19 pandemic, a fall in hospital admissions for child infections was observed in England due to societal strategy changes (social distancing, minimal recreational activities, restricted travel) and behavioral changes (adoption of non-pharmacological interventions) [37]. These changes could have reduced admissions for severe iGAS infections in adults. However, we report an increased incidence of ICU admission for severe iGAS in our study.

The potentiality that a change in the organization, ICU practice, or staff could have affected the results was low despite not being excluded. Indeed, the number of admissions in all ICUs remained similar before and after the COVID-19 pandemic, as were the timing of care and ICU practices (surgical intervention, norepinephrine, and RRT use).

Clindamycin, linezolid, and IVIG are the only available antitoxin drugs. In this study, we observed an association between clindamycin use and decreased ICU mortality in STSS patients. Clinical data are lacking to get beyond in vitro studies, with only one comparative observational study suggesting a potential reduction when clindamycin is added to penicillin, especially if administered with IGIV [38]. Our study has the same limitations; in particular, we cannot rule out selective survival bias even if most STSS patients received clindamycin within 24 h of ICU admission (59/84 patients, 71%).

Our study has other limitations. First, the before–after design can induce a bias in the systematic diagnosis of iGAS infection and under-diagnosis of viral co-infection, especially before COVID-19 pandemic. Pathophysiological data strongly suggest a role for viral-induced immune suppression in the onset of severe iGAS infection. This point should be further assessed in large prospective cohorts. Second, despite being one of the largest cohorts in the field and involving nearly 40 ICUs all over France, this study could lead to an inadequate estimate of cases because it does not fully represent the broader population (selection bias). It can be hypothesized that an over-estimating effect if centers facing an increase in iGAS cases were more prone to participate or an under-estimating effect if some reference centers declined to participate. Furthermore, using ICD code to identify cases has limitations, notably missed cases that were not assigned the codes and misassigned cases. The lack of serotyping studies on the samples prevents obtaining more insights into the specific strains of iGAS involved. Moreover, this is a retrospective study without control cases, which does not allow any causal link.

## Conclusion

The incidence of iGAS infections requiring ICU admission increased by 4 to 5-fold after the COVID-19 pandemic. The rate of STSS was higher in iGAS patients after the pandemic, with more frequent requests for IMV but no significant increase in ICU mortality rate. In iGAS patients, IMV, STSS, AKI, immunosuppression, and diabetes were significantly associated with ICU mortality. Further studies are needed to enhance our understanding of pathophysiology of GAS diseases and to explore causes of recent resurgence of severe iGAS infections. New therapeutics are still to be discovered to improve STSS patient care.

## Take-home message

- The incidence of iGAS infections requiring ICU admission by admission increased by 4 to 5-fold after the COVID-19 pandemic.
- After the COVID-19 pandemic, the rate of STSS was higher, with no significant increase in ICU mortality rate.

### Supplementary Information

**Additional file 1.** Supplemental Methods including STSS definition and detailed statistical analysis.

**Additional file 2.** Supplemental Figures and Tables.

## Data Availability

The data supporting the findings of the study are available upon reasonable request after approval of a proposal from the corresponding author.

## Abbreviations

AKI: Acute kidney injury
CKD: Chronic kidney disease
CNIL: French National Commission on Informatics and Liberty
COPD: Chronic obstructive pulmonary disease
eGFR: Estimated glomerular filtration rate
GAS: Group A *Streptococcus*
HR: Hazard ratio
ICU: Intensive care unit
iGAS: Invasive group A streptococcal
IMV: Invasive mechanical ventilation
IQR: Interquartile range
ISTRE: Infections invasives à STreptocoque du groupe A en RÉanimation
IVIG: Intravenous immunoglobulin
LRTI: Lower respiratory tract infections
NSAID: Non-steroidal anti-inflammatory drugs
RRT: Renal replacement therapy
RSV: Respiratory syncytial virus
SD: Standard deviation
SOFA: Sequential Organ Failure Assessment
SSTI: Skin and soft tissue infections
STSS: Streptococcal toxic shock-like syndrome
VA-ECMO: Veno-arterial extracorporeal membrane oxygenation
WHO: World Health Organization
