# Supplementary material for: Invasive group A streptococcal infections requiring admission to ICU: a nationwide, multicenter, retrospective study (ISTRE study)
Source: Crit Care. 2024 Jan 2;28:4. doi: 10.1186/s13054-023-04774-2 (PMC10759709; doi:10.1186/s13054-023-04774-2)

***Supplementary Information***

***Supplemental Methods***

**STSS definition:**

STSS was defined by a hypotension defined by a systolic blood pressure less than or equal to 90 mm Hg and multi-organ involvement characterized by two or more of the following:

- Renal impairment: Creatinine greater than or equal to 177 µmol/L or in patients with preexisting renal disease, a greater than twofold elevation over the baseline level.
- Coagulopathy: Platelets less than or equal to 100,000/mm^3^ or disseminated intravascular coagulation, defined by prolonged clotting times, low fibrinogen level, and the presence of fibrin degradation products.
- Liver involvement: Alanine aminotransferase, aspartate aminotransferase, or total bilirubin levels greater than or equal to twice the upper limit of normal for the patient's age. In patients with preexisting liver disease, a greater than twofold increase over the baseline level.
- Acute respiratory distress syndrome: defined by acute onset of diffuse pulmonary infiltrates and hypoxemia in the absence of cardiac failure or by evidence of diffuse capillary leak manifested by acute onset of generalized edema, or pleural or peritoneal effusions with hypoalbuminemia.
- A generalized erythematous macular rash that may desquamate. Soft-tissue necrosis, including necrotizing fasciitis or myositis, or gangrene.

***“Goodness of fit” of regression analysis:***

Table 3. Factors associated with ICU mortality for iGAS infections

| AIC | BIC | R^2^N |
| --- | --- | --- |
| 173 | 194 | 0.361 |

Table S3. Factors associated with ICU mortality for STSS patients

| AIC | BIC | R^2^N |
| --- | --- | --- |
| 138 | 155 | 0.369 |

*AIC: Akaike’s Information Criteria; BIC: Bayesian information criteria; R^2^N: Nagelkerke R Squared*

***Multicollinearity detection with the variance inflation factor (VIF):***

Table 3. Factors associated with ICU mortality for iGAS infections

|  | VIF |
| --- | --- |
| Diabetes | 1.17 |
| Immunosuppression | 1.06 |
| STSS | 1.14 |
| Invasive mechanical ventilation | 1.18 |
| Acute kidney injury | 1.03 |

 Table S3. Factors associated with ICU mortality for STSS patients

|  | VIF |
| --- | --- |
| Diabetes | 1.22 |
| Immunosuppression | 1.03 |
| Clindamycin | 1.09 |
| Invasive mechanical ventilation | 1.23 |
| Acute kidney injury | 1.03 |

*STSS: streptococcal toxic shock syndrome; VIF: variance inflation factor*

***Proportional hazard assumption tests (Cox analysis) with log(−log) plots and scaled Schoenfeld residuals:***

**Figure 2. Comparison of survival rates according to time from iGAS infection with and without streptococcal toxic shock syndrome (STSS)**

|  | Chisq | df | p |
| --- | --- | --- | --- |
| STSS | 0.63 | 1 | 0.43 |


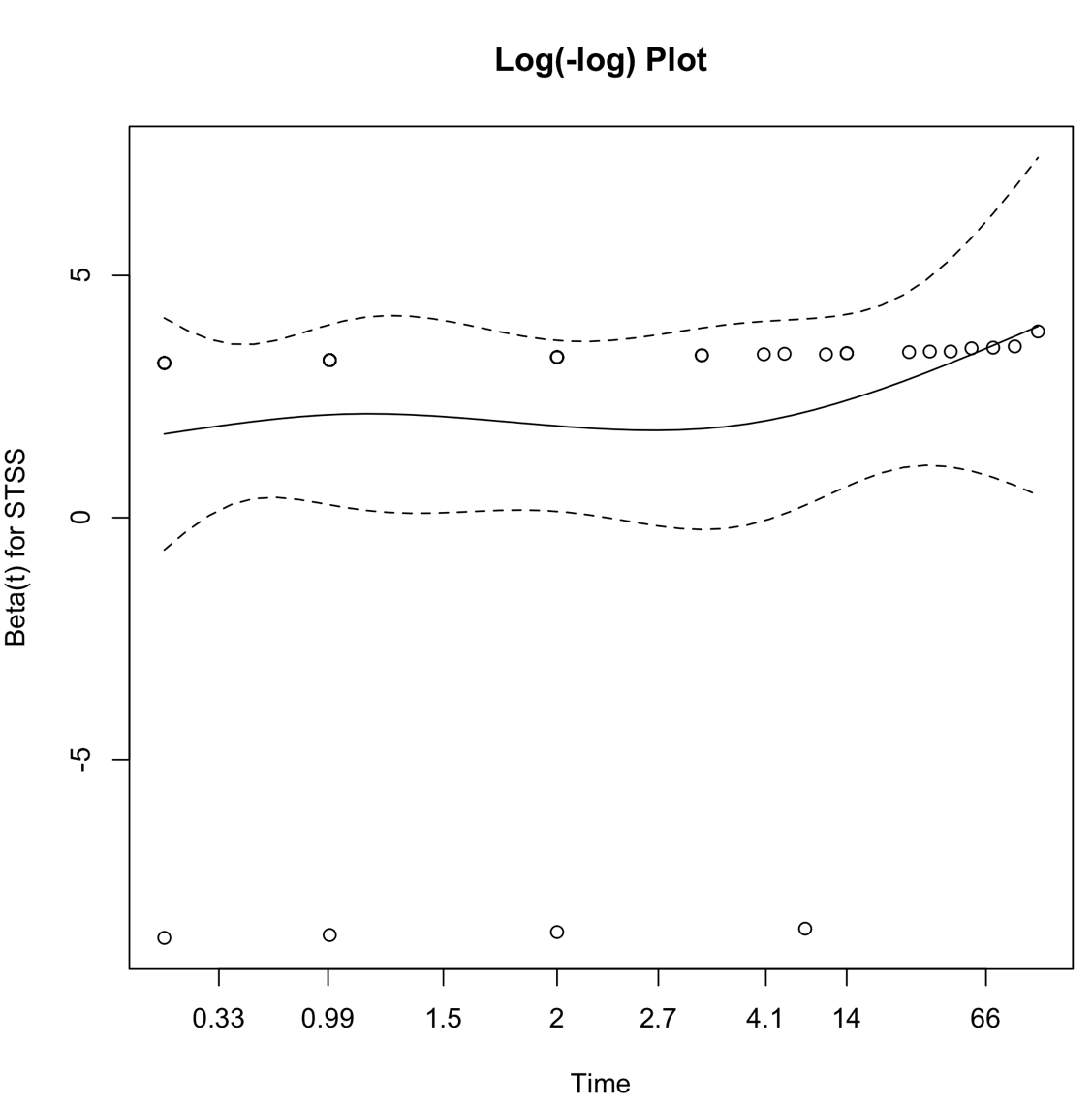

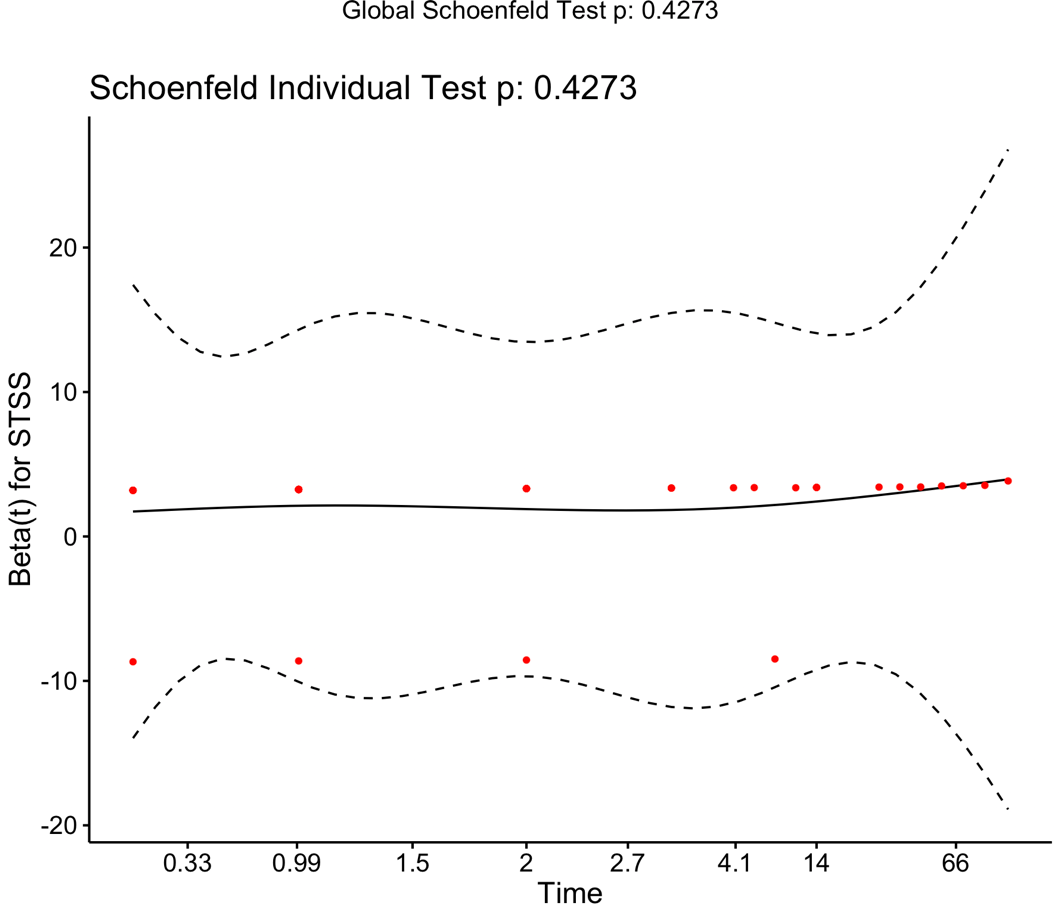


**Figure S2. Comparison of survival rates according to time from iGAS infection patients before and after COVID-19**

|  | Chisq | df | p |
| --- | --- | --- | --- |
| Period | 0.015 | 1 | 0.9 |


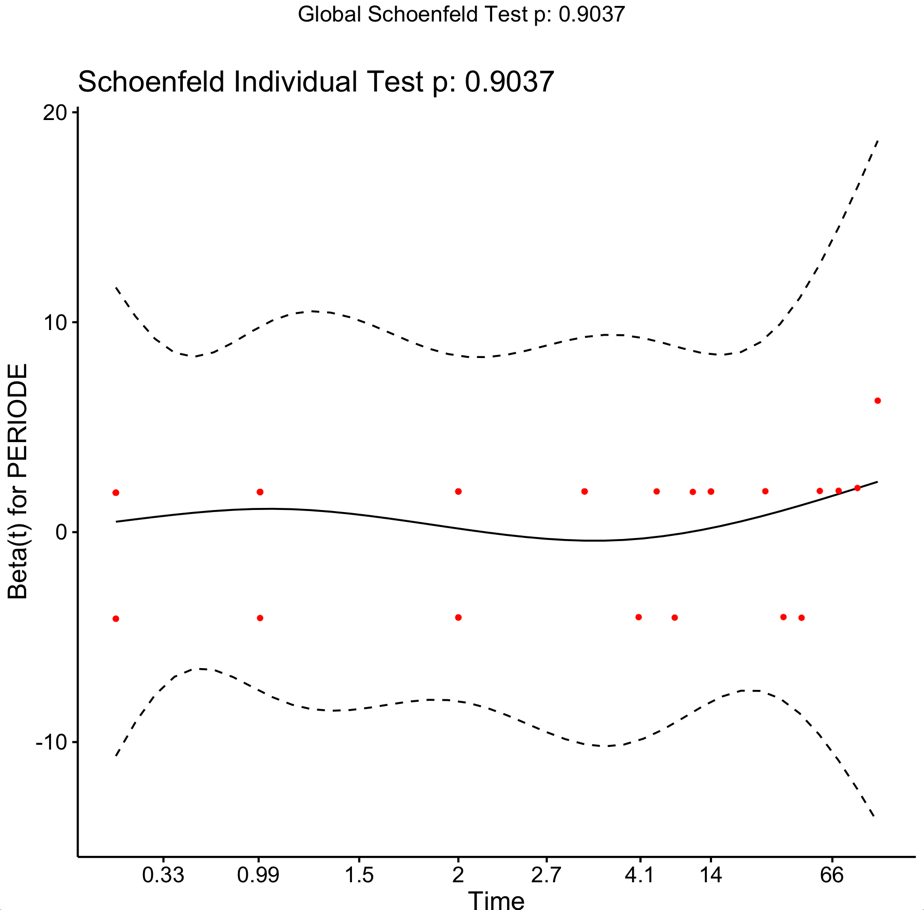

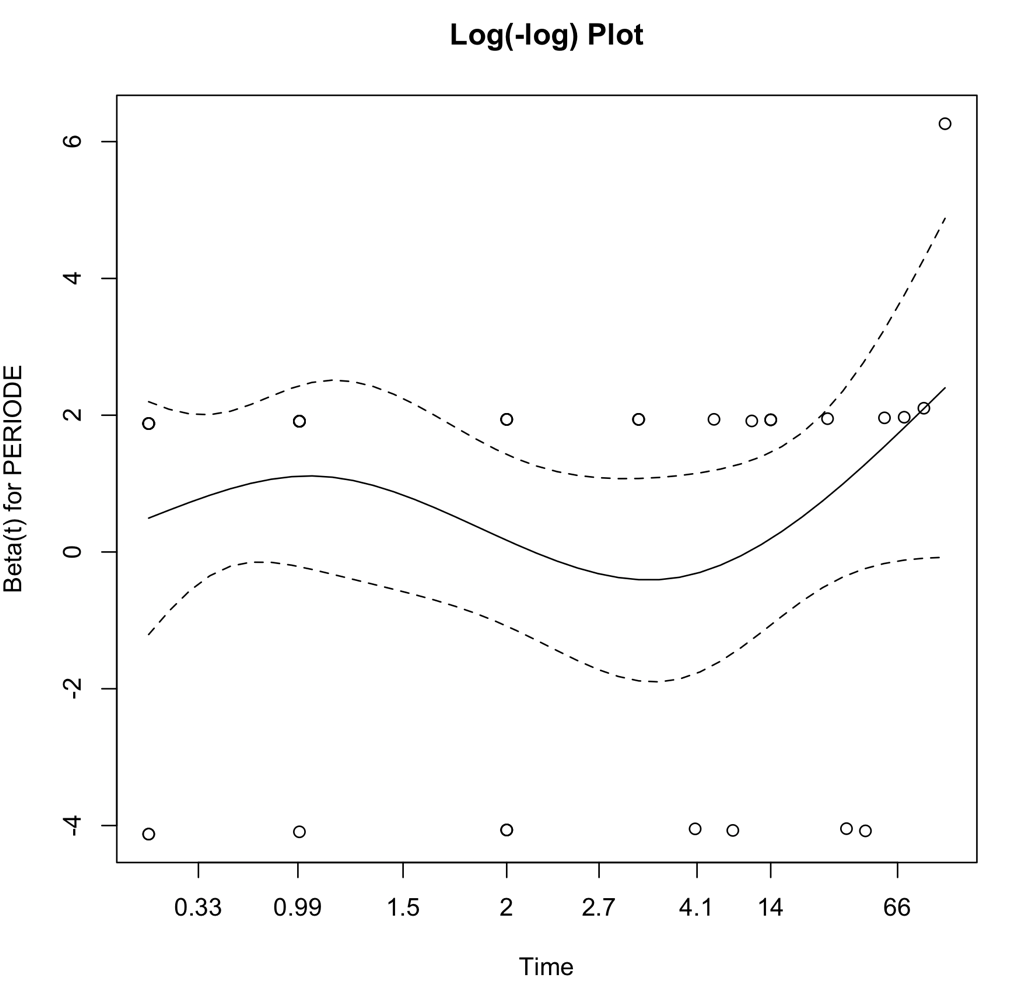

Supplement: Supplementary file 1 — Additional file 1. Supplemental Methods including STSS definition and detailed statistical analysis. [file 13054_2023_4774_MOESM1_ESM.docx]
