# Supplementary material for: Invasive group A streptococcal infections requiring admission to ICU: a nationwide, multicenter, retrospective study (ISTRE study)
Source: Crit Care. 2024 Jan 2;28:4. doi: 10.1186/s13054-023-04774-2 (PMC10759709; doi:10.1186/s13054-023-04774-2)
Supplement: Supplementary file 2 — Additional file 2. Supplemental Figures and Tables. [file 13054_2023_4774_MOESM2_ESM.docx]

***Supplementary Information***

| 1 | Alès |
| --- | --- |
| 2 | Angoulême |
| 3 | Annecy |
| 4 | Bordeaux (Thoracic ICU) |
| 5 | Bordeaux (Medical ICU) |
| 6 | Boulogne sur Mer |
| 7 | Caen |
| 8 | Cholet |
| 9 | Contamine-sur-Arve |
| 10 | Dax |
| 11 | Dieppe |
| 12 | Dunkerque |
| 13 | Étampes |
| 14 | Haguenau |
| 15 | La-Roche-sur-Yon |
| 16 | La Rochelle |
| 17 | Le Havre |
| 18 | Le Mans |
| 19 | Lens |
| 20 | Libourne |
| 21 | Longjumeau |
| 22 | Lorient |
| 23 | Meulan |
| 24 | Mulhouse |
| 25 | Nantes |
| 26 | Orléans |
| 27 | Pau |
| 28 | Périgueux |
| 29 | Roanne |
| 30 | Saintes |
| 31 | Saint Brieuc |
| 32 | Saint Nazaire |
| 33 | Saint Lô |
| 34 | Toulon |
| 35 | Tours |
| 36 | Vannes |
| 37 | Villejuif |

**Figure S1. Participating ICUs in metropolitan France**

**
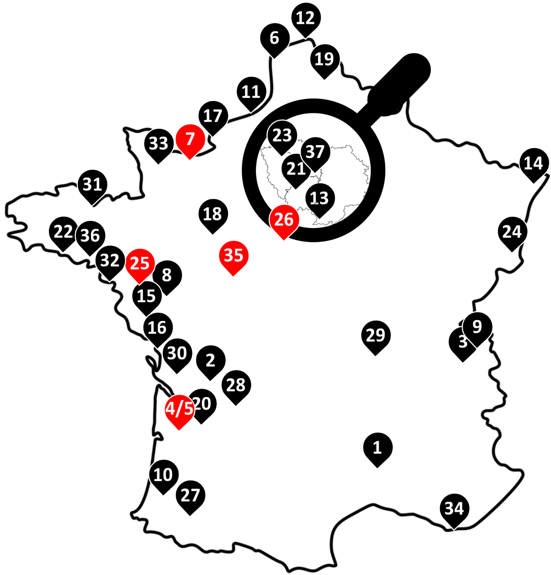
**

*Red icon: university hospital*


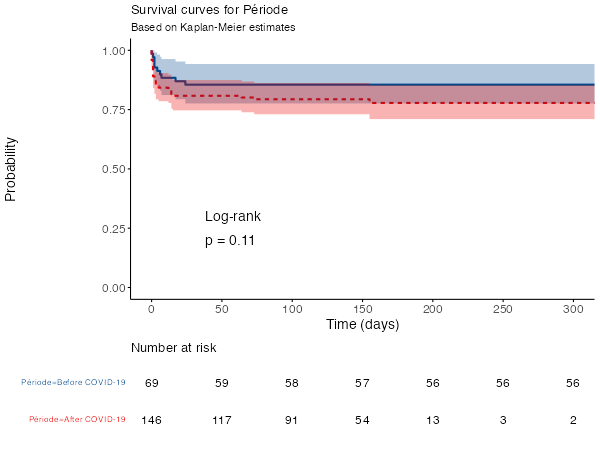
*Black icon: non-university hospita*


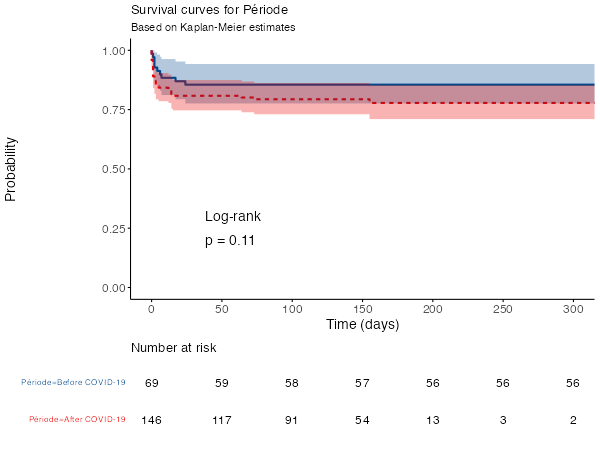
**Figure S2. Comparison of survival rates according to time from iGAS infection patients before and after COVID-19**

**Table S1. Characteristics of ICUs and case rate according study period**

Case rate > 1000: red

Case rate 500-1000: orange

Case rate <500: green

Before COVID-19 included two 6-month periods (October 1^st^ to March 31^st^) in 2018-2019 and 2019-2020.

After COVID-19 included one 6-month period (October 1^st^ to March 31^st^) in 2022-2023.

Case rate or person-time rate (number of incident events divided by the cumulative at-risk time in the sample: a time-constant incidence hazard): number of iGAS infections during period of interest divided by 100.000 ICU admissions during period of interest.

| **Demographic variables** | | | **Total ICU admissions** | | **iGAS infections** | | **Case rate** | |
| --- | --- | --- | --- | --- | --- | --- | --- | --- |
| **N°** | **City** | **Hospital** | **Before COVID-19** | **After COVID-19** | **Before COVID-19** | **After COVID-19** | **Before COVID-19** | **After COVID-19** |
| 1 | Alès | Non-university | 298 | 137 | 3 | 5 | 1006.71 | 3649.64 |
| 2 | Angoulême | Non-university | 504 | 238 | 0 | 2 | 0 | 840.34 |
| 3 | Annecy | Non-university | 1,055 | 463 | 3 | 8 | 284.36 | 1727.86 |
| 4 | Bordeaux (Thoracic ICU) | University | 559 | 230 | 2 | 1 | 357.78 | 434.78 |
| 5 | Bordeaux (Medical ICU) | University | 3,284 | 1,622 | 7 | 17 | 213.15 | 1048.09 |
| 6 | Boulogne sur Mer | Non-university | 713 | 404 | 4 | 5 | 561.01 | 1237.62 |
| 7 | Caen | University | 839 | 343 | 2 | 2 | 238.38 | 583.09 |
| 8 | Cholet | Non-university | 742 | 392 | 0 | 2 | 0 | 510.20 |
| 9 | Contamine-sur-Arve | Non-university | 489 | 337 | 0 | 6 | 0 | 1780.42 |
| 10 | Dax | Non-university | 483 | 263 | 0 | 2 | 0 | 760.46 |
| 11 | Dieppe | Non-university | 729 | 366 | 0 | 2 | 0 | 546.44 |
| 12 | Dunkerque | Non-university | 717 | 335 | 1 | 5 | 139.47 | 1492.54 |
| 13 | Étampes | Non-university | 744 | 241 | 2 | 3 | 268.82 | 1244.81 |
| 14 | Haguenau | Non-university | 635 | 310 | 1 | 0 | 157.48 | 0 |
| 15 | La-Roche-sur-Yon | Non-university | 1,008 | 512 | 1 | 11 | 99.21 | 2148.43 |
| 16 | La Rochelle | Non-university | 598 | 278 | 4 | 2 | 668.90 | 719.42 |
| 17 | Le Havre | Non-university | 1,011 | 437 | 2 | 5 | 197.82 | 1144.16 |
| 18 | Le Mans | Non-university | 1,578 | 795 | 4 | 5 | 253.49 | 628.93 |
| 19 | Lens | Non-university | 693 | 297 | 2 | 1 | 288.60 | 336.70 |
| 20 | Libourne | Non-university | 868 | 508 | 0 | 4 | 0 | 787.40 |
| 21 | Longjumeau | Non-university | 579 | 291 | 1 | 3 | 172.71 | 1030.93 |
| 22 | Lorient | Non-university | 1,214 | 483 | 2 | 5 | 164.75 | 1035.20 |
| 23 | Meulan | Non-university | 420 | 170 | 0 | 1 | 0 | 588.24 |
| 24 | Mulhouse | Non-university | 866 | 310 | 2 | 3 | 230.95 | 967.74 |
| 25 | Nantes | University | 2,092 | 796 | 9 | 7 | 430.21 | 1256.28 |
| 26 | Orléans | University | 1,278 | 525 | 1 | 4 | 78.25 | 761.90 |
| 27 | Pau | Non-university | 956 | 398 | 2 | 5 | 209.20 | 1256.28 |
| 28 | Périgueux | Non-university | 839 | 331 | 4 | 4 | 476.76 | 1208.46 |
| 29 | Roanne | Non-university | 444 | 200 | 1 | 1 | 225.23 | 500 |
| 30 | Saintes | Non-university | 669 | 342 | 1 | 4 | 149.47 | 1169.59 |
| 31 | Saint Brieuc | Non-university | 1,045 | 576 | 2 | 8 | 191.39 | 1388.89 |
| 32 | Saint Nazaire | Non-university | 542 | 294 | 1 | 0 | 184.51 | 0 |
| 33 | Saint Lô | Non-university | 600 | 254 | 1 | 1 | 166.67 | 393.70 |
| 34 | Toulon | Non-university | 1,811 | 639 | 1 | 5 | 55.22 | 782.47 |
| 35 | Tours | University | 2,723 | 951 | 4 | 2 | 146.90 | 210.30 |
| 36 | Vannes | Non-university | 1,403 | 674 | 3 | 7 | 213.83 | 1038.58 |
| 37 | Villejuif | Non-university | 582 | 221 | 0 | 1 | 0 | 452.49 |
| **Total** | | | **35,610** | **15,963** | **73** | **149** | **205** | **949** |

**Table S2. Factors associated with ICU mortality for iGAS infections**

|  | **Univariate analysis** | | |
| --- | --- | --- | --- |
| **Variables** | **OR** | **CI95%** | **p-value** |
| Period (after vs before COVID-19 pandemic) | 1.79 | 0.83-3.87 | 0.138 |
| Male sex | 1.15 | 0.59-2.28 | 0.678 |
| Age (per year) | 1.03 | 1.01-1.06 | 0.005 |
| Hypertension | 1.20 | 0.61-2.39 | 0.595 |
| Diabetes | 2.23 | 1.01-4.91 | 0.047 |
| Coronary disease | 0.86 | 0.28-2.69 | 0.800 |
| Immunosuppression | 2.74 | 0.94-8.01 | 0.066 |
| COPD | 1.27 | 0.33-4.82 | 0.728 |
| Chronic kidney disease | 1.43 | 0.15-3.15 | 0.621 |
| Influenza co-infection | 1.41 | 0.59-3.38 | 0.444 |
| Infection site |  |  |  |
| *Skin and soft tissue infection* | 1 |  |  |
| *Lower respiratory tract infection* | 0.59 | 0.27-1.27 | 0.176 |
| *Others* | 0.51 | 0.20-1.28 | 0.158 |
| STSS | 11.03 | 3.78-32.14 | <0.001 |
| Monotherapy | 0.82 | 0.38-1.74 | 0.601 |
| Surgical source control | 0.74 | 0.37-1.49 | 0.401 |
| Clindamycin use | 0.79 | 0.41-1.54 | 0.491 |
| Linezolid use | 1.63 | 0.70-3.81 | 0.258 |
| Immunoglobulin use | 1.83 | 0.61-5.51 | 0.282 |
| Invasive mechanical ventilation | 8.43 | 2.89-24.58 | <0.001 |
| Dobutamine use | 2.88 | 1.16-7.11 | 0.022 |
| Acute kidney injury | 11.69 | 2.74-49.91 | <0.001 |
| Renal replacement therapy | 4.90 | 2.42-9.94 | <0.001 |

*COPD: chronic obstructive pulmonary disease; GAS: group A Streptoccocus; ICU: intensive care unit; STSS: streptococcal toxic shock syndrome*

*Univariate comparison was performed using linear logistic regression (all variables fit normal distribution).*

**Table S3. Factors associated with ICU mortality for STSS**

|  | **Univariate analysis** | | | **Multivariate analysis** | | |
| --- | --- | --- | --- | --- | --- | --- |
| **Variables** | **OR** | **CI95%** | **p-value** | **OR** | **CI95%** | **p-value** |
| Period (after vs before COVID-19 pandemic) | 1.26 | 0.52-3.05 | 0.613 |  |  |  |
| Male sex | 1.41 | 0.64-3.08 | 0.394 |  |  |  |
| Age (per year) | 1.05 | 1.01-1.08 | 0.003 |  |  |  |
| Hypertension | 1.63 | 0.75-3.56 | 0.220 |  |  |  |
| **Diabetes** | 3.27 | 1.23-8.74 | 0.018 | **8.79** | **2.26-34.21** | **0.002** |
| Coronary disease | 1.01 | 0.26-4.58 | 0.913 |  |  |  |
| Immunosuppression | 3.77 | 0.84-17.55 | 0.102 | 5.08 | 0.88-29.35 | 0.070 |
| COPD | 2.25 | 0.43-11.69 | 0.335 |  |  |  |
| Chronic kidney disease | 0.41 | 0.05-3.68 | 0.430 |  |  |  |
| Influenza co-infection | 1.01 | 0.37-2.71 | 0.990 |  |  |  |
| Infection site |  |  |  |  |  |  |
| *Skin and soft tissue infection* | 1 |  |  |  |  |  |
| *Lower respiratory tract infection* | 0.59 | 0.29-1.31 | 0.109 |  |  |  |
| *Others* | 0.39 | 0.19-1.25 | 0.102 |  |  |  |
| Monotherapy | 1.19 | 0.46-3.01 | 0.541 |  |  |  |
| Surgical source control | 0.66 | 0.30-1.42 | 0.285 |  |  |  |
| **Clindamycin use** | 0.43 | 0.19-0.96 | 0.022 | **0.20** | **0.08-0.54** | **0.002** |
| Linezolid use | 1.03 | 0.42-2.53 | 0.955 |  |  |  |
| Immunoglobulin use | 0.98 | 0.31-3.03 | 0.966 |  |  |  |
| **Invasive mechanical ventilation** | 5.41 | 1.19-24.52 | 0.029 | **19.60** | **2.52-152.53** | **0.005** |
| Dobutamine use | 1.78 | 0.68-4.65 | 0.243 |  |  |  |
| **Acute kidney injury** | 8.94 | 1.14-70.07 | 0.037 | **11.58** | **1.34-99.78** | **0.026** |
| Renal replacement therapy | 3.39 | 1.54-7.48 | 0.002 |  |  |  |

***Statistical significance (p<0.05)***

*GAS: group A Streptoccocus; ICU: intensive care unit; NSAID: non-steroidal anti-inflammatory drug; STSS: streptococcal toxic shock syndrome*

*Univariate comparison was performed using linear logistic regression. Independent variables with a p-value < 0.20 were considered for inclusion in the multivariate analysis using linear logistic regression. We excluded “renal replacement therapy” variable due to collinearity with acute kidney injury. We applied a backward stepwise selection for age, diabetes, immunosuppression, infection site, clindamycin use, invasive mechanical ventilation, and acute kidney injury to identify factors significantly associated with ICU mortality in STSS patients. The ratio was of one independent variable for 8 events.*
